# Stimuli-Responsive Polymers Prevent Severe Hypoglycemia

**DOI:** 10.1021/acscentsci.4c01834

**Published:** 2024-11-07

**Authors:** Kaden C. Stevens, Brent S. Sumerlin

**Affiliations:** George & Josephine Butler Polymer Research Laboratory, Department of Chemistry, Center for Macromolecular Science & Engineering, University of Florida, Gainesville, Florida 32611, United States

Exogenous insulin injection is a common, effective treatment for diabetic patients that works by reducing blood glucose levels during periods of hyperglycemia. Unfortunately, insulin treatments can also reduce blood glucose to dangerously low levels, resulting in life-threatening hypoglycemia. Currently, severe hypoglycemia is treated by injections of glucose and glucagon (GCG), a peptide that works to increase native glucose production within the body. Although effective, these treatments are reactive and not preventative. In this issue of *ACS Central Science*, Hevener, Maynard, and co-workers address this problem by creating glucose-responsive micelles that autonomously release GCG at dangerously low glucose levels to treat and avoid deep hypoglycemia.^1^

**Figure 1:**
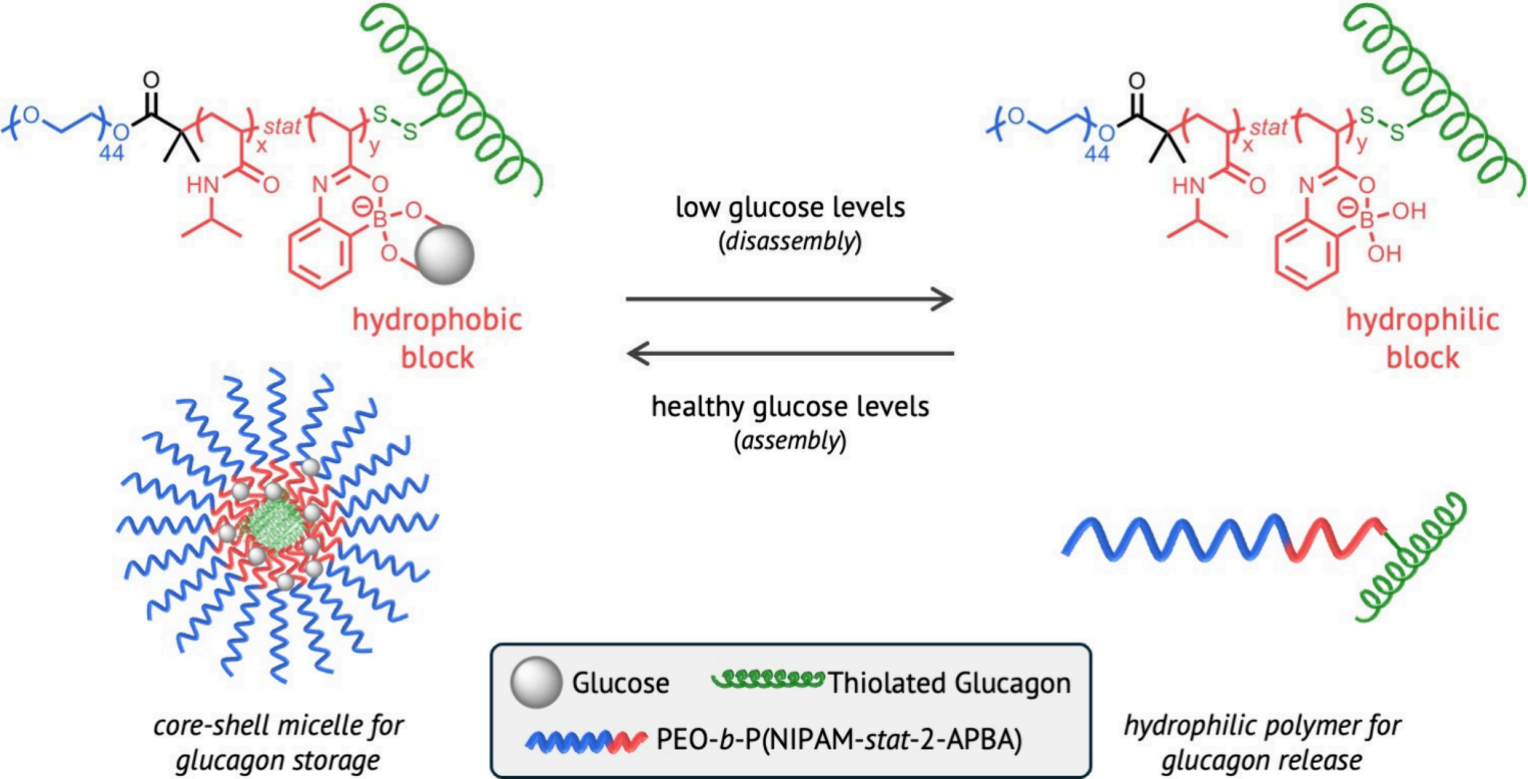
Proposed mechanism of glucose-responsive self-assembly for glucagon release.

Stimuli-responsive polymer self-assembly is generally accomplished by synthesizing polymers with chemically distinct segments along the backbone, called block copolymers, where one or more of the blocks are embedded with stimuli-responsive pendent moieties.^2^ After dissolution, external stimuli such as light, temperature, pH, etc. can be used to alter the solubility of a responsive block, thereby inducing solvent-selective assembly of the block copolymers on demand.^3^ The Maynard team used glucose levels as the external stimulus by incorporating boronic acid pendent groups that reversibly bind with sugar. The block copolymers were comprised of a glucose-agnostic corona-forming block that remained hydrophilic and a glucose-responsive boronic acid-containing block with a GCG unit attached to its terminus (Figure 1).^4,5^ At normal glucose levels, equilibrium drives the association of glucose with the boronic acid units, rendering the boronic-acid-containing block hydrophobic and driving assembly of the block copolymer into core–shell micelles. Because the GCG is attached at the terminus of the core-forming block, it is buried within the assembly at normal glucose levels, limiting its slow background release. Once the micelles are exposed to low glucose environments, the equilibrium shifts to release glucose and expose free boronic acid moieties that induce a transition in solubility. With both segments of the block copolymer then being hydrophilic, the micelles dissociate and reveal the GCG end groups that act to prevent or reverse deep hypoglycemia.

**Figure 2:**
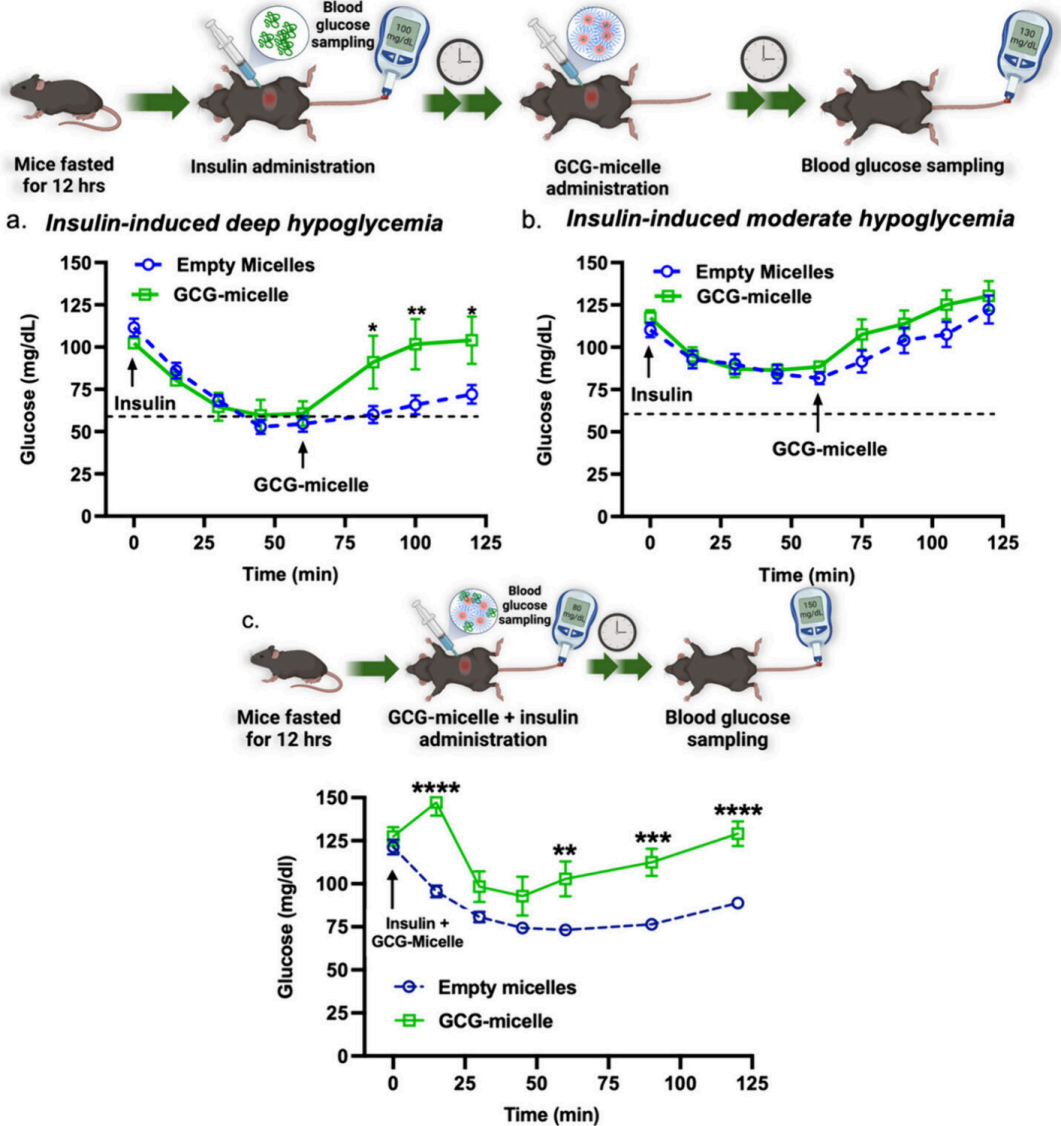
Glucose recovery after deep (a) or mild (b) hypoglycemia. The assembly/disassembly threshold is represented by a dotted line. (c) Prevention of hypoglycemia after GCG-micelle and insulin co-administration. Glucose levels are represented as the mean value ± SEM for *n* = 5–6. Statistical comparison of the different glucose levels between the two groups at equivalent times represented with *p* < 0.05 (*), *p* < 0.01 (**), *p* < 0.001 (***), *p* < 0.0001 (****). Reproduced with permission from ref (1). Copyright 2024 American Chemical Society.

In vivo experiments demonstrated the efficacy of GCG-micelles in addressing and preventing hypoglycemia. In the first scenario, hypoglycemia was induced in fasted mice via insulin injection, followed 60 min later by an injection of GCG-micelles (Figure 2A and B). Micelle injection led to a significantly faster rise in glucose levels for the deeply hypoglycemic group relative to a control group (Figure 2A), while the glucose levels of the moderately hypoglycemic mice with and without the GCG-micelle injections were statistically indistinguishable from the control (Figure 2B). These results suggest that GCG-micelles only release a significant quantity of GCG in deeply hypoglycemic environments. In a separate study, the team demonstrated the efficacy of GCG-micelles as a preventative therapeutic by simultaneous administration with a dose of insulin large enough to induce deep hypoglycemia. In this case, GCG-micelles prevented the onset of hypoglycemia and led to a more rapid recovery of blood sugar relative to the control group. The authors go to great lengths to demonstrate the safety profile of the GCG-micelles to ensure regular co-injection with exogenous insulin would avoid unwanted side effects. If insulin administration lowers blood glucose to dangerous levels, the GCG-micelles activate to prevent severe hypoglycemia. If an insulin injection lowers blood glucose to a healthy range, the micelles remain stable and inactive.

As the authors acknowledge, this proof-of-concept will benefit by addressing a few key challenges before reaching clinical significance. Most importantly, while the stability, safety, and effectiveness of these GCG-micelle designs have been thoroughly characterized in mice, validation in additional animal models and human studies can address any differences in glucose dynamics between species.^6^ Moreover, the synthetic versatility of the approach suggests that additional block copolymer examples could be readily prepared to further investigate the fundamental structure-pharmacokinetic relationships, including long-term stability or strategies for extending the duration of action.^7^

This report lays the groundwork for the continued development of non-toxic, autonomous, and preventative treatments that can be co-delivered with insulin to mitigate the most dangerous symptoms of hypoglycemia. This basic strategy represents a significant innovation that could dramatically improve the safety and efficacy of symptom management for the millions of people worldwide with diabetes.
